# Hospitalised older adults with community-acquired pneumonia and sepsis have dysregulated neutrophil function but preserved glycolysis

**DOI:** 10.1136/thorax-2024-222215

**Published:** 2024-12-16

**Authors:** Frances Grudzinska, Aduragbemi A Faniyi, Kylie B R Belchamber, Celine Chen, Robert Stockley, Alice Jasper, Dhruv Parekh, Elizabeth Sapey, Aaron Scott, David R Thickett

**Affiliations:** 1 Birmingham Acute Care Research Group, Institute of Inflammation and Ageing, University of Birmingham, Birmingham, UK; 2 School of Translational Medicine, University of Nottingham, Nottingham, UK; 3 NIHR Birmingham Biomedical Research Centre, Birmingham, UK; 4 The University of Manchester Maternal and Fetal Health Research Centre, Manchester, UK; 5 Institute of Inflammation and Ageing, University of Birmingham, Birmingham, UK; 6 NIHR Birmingham Clinical Research Facility, Birmingham, UK; 7 PIONEER HDR-UK Hub in Acute Care, Birmingham, UK

**Keywords:** Pneumonia, Neutrophil Biology, Innate Immunity, Respiratory Infection

## Abstract

**ABSTRACT:**

**Objective:**

Community-acquired pneumonia (CAP) is a leading cause of hospitalisation in older adults and is associated with a high likelihood of adverse outcomes. Given the ageing population and lack of therapeutic advances in CAP, new strategies to manage the burden of this disease are needed. Neutrophil dysfunction has been widely demonstrated in CAP and is associated with poor outcomes. We hypothesised that impaired glycolytic metabolism was driving neutrophil dysfunction in older adults with CAP.

**Methods:**

To investigate the mechanism underlying neutrophil dysfunction in CAP, we recruited older adults with CAP and sepsis, age-matched controls and healthy young adults to assess neutrophil function and glycolytic metabolism in peripheral blood neutrophils.

**Results:**

We demonstrate that neutrophils from older donors with CAP display a broad range of functional defects, including inaccurate migration to interleukin 8, impaired respiratory burst in response to phorbol 12-myristate 13-acetate and increased spontaneous degranulation compared with age-matched controls. Glycolysis (assessed by extracellular flux and RNA-sequencing) was not significantly altered between age-matched groups; however, basal rates of neutrophil glycolysis were significantly higher in patients with CAP and older adult controls compared with healthy young adults, and stimulated glycolysis was significantly higher in young adults compared with older adults with and without CAP.

**Conclusions:**

Our findings suggest that neutrophil dysfunction in older adults with CAP may be implicated in poor outcomes, irrespective of glycolytic metabolism.

**WHAT IS ALREADY KNOWN ON THIS TOPIC**

- Neutrophils from donors with pneumonia, and in particular older adults with pneumonia display a broad range of defects; however, the mechanism driving aberrant function is unknown.

**WHAT THIS STUDY ADDS**

- We demonstrate impaired neutrophil functions during pneumonia and sepsis but show that glycolysis assessed both directly and by gene expression is not the driver of dysfunction.

**HOW THIS STUDY MIGHT AFFECT RESEARCH, PRACTICE OR POLICY**

- This work highlights the importance of investigating immune cell defects in the population at risk. Other studies with younger participants who are critically ill due to infection have implicated immunometabolism in innate immune cell dysfunction; however, we demonstrate in older adults that neutrophil glycolysis is unchanged during pneumonia.

## Introduction

 Community-acquired pneumonia (CAP) significantly impacts older adults, leading to emergency hospitalisation, high mortality and readmission rates. Studies report 24% 30-day mortality in adults ≥60 years with 1 year mortality rising to 47%,^1^ readmission within 30 days occurs in 16%,^2^ older adults also report a significant burden of ill health following CAP.^3^ The high prevalence of adverse outcomes in older adults highlights the urgent need for innovative management strategies to address these issues.^4^

Neutrophils are a core part of the innate immune response to infection; however, neutrophil responses must balance pro-inflammatory actions to control infection while limiting bystander healthy tissue damage. The importance of this balance is emphasised by the poor outcomes seen in patients with specific neutrophil defects which inhibit neutrophil functions^5^ and development of acute respiratory distress syndrome in the presence of excessive neutrophilic inflammation.^6^

Neutrophil function is impaired with age. Neutrophil migratory accuracy is reduced with increasing age and frailty^7 8^ as is both the phagocytosis of opsonised *Escherichia coli*^9^ and the generation of neutrophil extracellular traps (NETosis).^10^ The aberrant neutrophil function seen in frail older adults is further impaired by pulmonary infection, where increased severity of infection is associated with increasing impairment of neutrophil function.^11^ There are associations between these altered neutrophil functions and patient outcomes, including increased mortality and complications in acute respiratory distress syndrome and sepsis.^12 13^

Immunometabolism is the relationship between cellular metabolism and function. This was first studied in macrophages, where metabolism influences cytokine production^14^ and has now been linked to other effector functions such as cellular migration across multiple immune cells.^15^ There is a paucity of data concerning neutrophil metabolism in health or disease states.

Neutrophils are predominantly glycolytic^16^ possessing mature mitochondria which are important for purinergic signalling and control of apoptosis^17^ but do not significantly contribute to ATP balance.^18^ Neutrophil functions are highly energy demanding,^16^ and increased expression of glycolytic genes has been demonstrated during infection.^19 20^ Neutrophils from donors with chronic obstructive pulmonary disease have lower levels of ATP than healthy controls.^21^ There are no data examining neutrophil metabolism in CAP or healthy older adults. Given previous data demonstrating a range of inhibited neutrophil functions in donors with infection^11 12^ and that neutrophil functions require glycolysis, we hypothesised that an alteration in neutrophil glycolysis might be responsible for the deficits in cell function.

The aim of this study was to establish the impact of pneumonia with sepsis on neutrophil effector functions in older adults and assess glycolytic metabolism.

## Materials and methods

### Study subjects

This was a single-centre prospective observational study conducted between November 2019 and August 2023.^22^ Participants with CAP met British Thoracic Society definition for CAP^23^ and sepsis and were ≥65 years of age. Sepsis was defined as a Quick Sequential Organ Failure Assessment (qSOFA) Score ≥2^24^ to provide a pragmatic means of assessing patients for sepsis outside critical care.^25^ Participants with CAP were recruited from the medical admissions unit within 36 hours of admission. Older adult controls were recruited from ophthalmology and healthcare of older persons outpatient clinics, they were matched to participants with CAP for age and clinical frailty score. Healthy young adults were recruited from the research staff. Full eligibility criteria and recruitment details are provided in the online supplementary data (online supplemental figure S1 and table 1). Usual care for CAP was delivered by the clinical team caring for patients.

### Isolation of blood neutrophils

Neutrophils were isolated from whole blood using a discontinuous Percoll gradient.^22^ Neutrophils were typically ≥97% pure, viability was assessed by Annexin V and propidium iodide staining (online supplemental methods).

### Neutrophil chemotaxis

Migration was assessed using an Insall Chamber and time-lapse microscopy which allows for parameters of migratory accuracy to be recorded.^22^ Coverslips were coated with 7.5% bovine serum albumin (Sigma-Aldrich) and 8×10^5^ neutrophils adhered for 20 min prior to inversion on to the Insall Chamber containing either 100 nM interleukin-8 or RPMI (negative control). The chemoattractant gradient was allowed to develop for 1 min prior to time-lapse microscopy using a Leica DMI 6000B microscope with a DFL350 FX camera. An image was captured every 20 s for 12 min yielding 37 images. Films were exported as AVI files and analysed by a single-blinded analyst using the manual tracking plugin on FIJI Image J (National Institutes of Health, Bethesda, Maryland, USA).^26^ These pixel position data were inputted into a preformatted Excel template^27^ to generate values for speed, velocity, chemotactic index, displacement, distance travelled and directness to describe cell movement. Chemotactic index is a measure of accuracy per frame where 1=movement directly towards the chemoattractant and −1=movement directly away from the chemoattractant. Directness is a measure of straightness of travel calculated by displacement (distance between start and end point irrespective of direction)/distance travelled over the whole time-lapse.^27^

### Neutrophil degranulation and plasma markers

Markers of degranulation were measured in plasma; Aα-Val541 a specific fibrinogen cleavage product used as a footprint of proteinase 3 (PR3) activity was measured by indirect ELISA.^28^ Aα-Val360 is a neutrophil elastase (NE) specific fibrinogen cleavage footprint measured by sandwich ELISA.^29^ Myeloperoxidase (MPO) and NE were measured using commercial kits (R&D Systems) according to the manufacturer’s instructions.

### Neutrophil oxidative burst

Neutrophil oxidative burst was assessed using the Seahorse Analyzer (Agilent Technologies). Neutrophils were treated with inhibitors of mitochondrial respiration (rotenone/anti-mycin A) followed by 160 nM phorbol 12-myristate 13-acetate (PMA) to generate a respiratory burst.^30^ Oxygen consumption was measured to assess oxidative burst over time.

### Neutrophil extracellular flux

Neutrophil metabolism was assessed using the Seahorse Analyzer quantifying extracellular acidification rate (ECAR) of media as a surrogate of lactate efflux. The assay involves use of both glycolytic and mitochondrial inhibitors to interrogate metabolic pathways. Freshly isolated neutrophils were seeded on a cell plate in phenol red and sodium bicarbonate free, filter sterilised RPMI (Merck Life Sciences, UK), supplemented with 2 mM l-glutamine, 1 mM sodium pyruvate, 25 mM glucose and 5 mM 4-(2-hydroxyethyl)-1-piperazineethanesulfonic acid (Merck Life Sciences, UK), pH 7.4. ECAR is measured over time with an injection of 160 nM PMA after measurement of baseline glycolysis, a subsequent injection of oligomycin inhibits mitochondrial activity to confirm that all measured activity is glycolytic in origin. Lastly, 2-deoxyglucose, an inhibitor of glycolysis, is added to confirm all metabolic activity is inhibited.^30^ ECAR is converted to proton efflux rate (PER) to account for the buffering capacity of media.^30^ Basal glycolysis is the last measurement prior to the addition of PMA, and fold change in glycolysis is measured after the addition of PMA relative to basal PER (online supplemental figure S2).

### Neutrophil cell surface receptors

Surface expression of cluster of differentiation (CD) 10, 11b,11c,16, 62L and 66b, programmed death-ligand 1 (PD-L1) and chemo-attractant receptors (CXCR) CXCR2 and CXCR4 were measured on freshly isolated peripheral blood neutrophils. Receptor expression was determined by flow cytometry using a MACSQuant 10 instrument (Miltenyi Biotec). Data were analysed using FlowJo software (BD Sciences). Mature neutrophils were defined as CD66b+/CD10+.^31^

### RNA expression

RNA isolation was performed as described previously.^22^ QuantSeq 3′ mRNA-Seq Library Prep Kit was used for cDNA library preparation from polyA+mRNA, followed by 75 bp, single-end sequencing using NextSeq550. Data processing is described in online supplemental methods and figure S3.

### Statistics

Normally distributed data were analysed using t-test. Mann-Whitney test was used for non-parametric data. Fisher’s exact test was used for categorical data. Analysis of experimental data was performed by GraphPad Prism, Dotmatics, USA (V.9.4.1). Demographic data were analysed using SPSS, IBM, USA (V.29.0). Missing data were excluded from analyses. P value <0.05 was considered significant. Adjustment for multiple comparisons was performed for RNAseq data using the DESeq2 package which uses the Benjamini-Hochberg procedure.^32^ Other data presented are not corrected for multiple comparisons.

Sample size calculation was based on detecting a significant difference in chemotactic index; Mean Chemotactic Index 0.30 (SD 0.13) in older adults without CAP and 62% reduction observed in pilot data in CAP with p<0.05 and 90% power indicating 10 participants in each arm to detect a difference in neutrophil chemotaxis. This limited sample size was not anticipated to inform relationships between clinical outcomes and neutrophil function.^22^

Due to experimental time constraints and access to equipment, not all assays were performed for neutrophils from all participants; therefore, over-recruitment was considered necessary to enable the assessment of function and glycolysis. Numbers of participants included in each analysis are listed in figure legends.

## Results

### Participant demographics and outcomes

57 participants were recruited. Participants with CAP and older adult controls were well matched for age, sex, ethnicity, clinical frailty score and major comorbidities known to influence neutrophil function (table 1). Controls were more likely to have received annual influenza and pneumococcal vaccination (p<0.001). Participants with CAP had a higher prevalence of dementia (p=0.032), were more likely to live in supported accommodation (p=0.005),and had poorer baseline mobility (p=0.027) and more domiciliary care (p=0.001).

**Table 1:** Baseline demographics of study participants

	Non-CAP control (n=32)	CAP(n=25)	P value
Age; mean (SD)	80.83 (7.03)	82.63 (8.16)	0.053
Female; number (%)	17 (53.1)	16 (64.0)	0.290
White ethnicity; number (%)	29 (90.6)	24 (96.0)	0.408
Never smoker; number (%)	18 (56.3)	12 (48.0)	0.082
Clinical frailty score; median (IQR)	4 (3–5)	5 (3–6)	0.341
Had pneumococcal vaccination; number (%)	20 (62.5)	11 (44.0)	**0.005**
Had Influenza vaccination; number (%)	29 (90.6)	14 (56.0)	**<0.0001**
BMI; mean (SD)	25.8 (4.2)	26.6 (6.2)	0.611
Number of comorbidities; median (IQR)	4 (2–6)	3 (2–4)	0.449
Cardiovascular disease; number (%)	18 (56.3)	16 (64.0)	0.067
Diabetes mellitus; number (%)	10 (31.3)	4 (16.0)	0.539
Dementia; number (%)	0	4 (16.0)	**0.032**
Number of medications (preadmission); median (IQR)	4 (2–6)	5 (3–7)	0.299
Antihypertensive drug; number (%)	18 (56.3)	16 (64.0)	0.597
Antiplatelet drug; number (%)	12 (37.5)	9 (36.0)	1.000
Anticoagulant drug; number (%)	3 (9.4)	3 (12.0)	1.000
Statin; number (%)	15 (46.9)	13 (52.0)	0.792
Oral diabetic drug; number (%)	10 (31.3)	4 (16.0)	0.227
Insulin; number (%)	6 (18.8)	0 (0.0)	**0.030**
Analgesia; number (%)	4 (12.5)	7 (28.0)	0.184
Usually resident in own home; number (%)	30 (93.8)	19 (76.0)	**0.005**
Independently mobile; number (%)	22 (68.8)	11 (44.0)	**0.027**
No domiciliary care; number (%)	30 (93.8)	14 (56.0)	**0.0003**

Fisher’s test was used for categorical data, Mann-Whitney test for non-parametric continuous data and Tt-test for parametric continuous data. P-values <0.05 are shown in bold.

BMI, Body Mass IndexCAP, community-acquired pneumonia

Participants with CAP were recruited a median of 26 hours following admission and had been symptomatic for a median of 3 days (IQR 1–4) prior to hospitalisation. All participants were negative for SARS-CoV2 and influenza. Microbiological testing rates were low with blood cultures sent in 56% and sputum cultures in 8% (online supplemental table 2). Urine testing for pneumococcal or legionella antigens was not performed in any participant. A microbiological cause was not identified in any participant. Patients had severe pneumonia with a median CURB65 score of 3 (IQR 3–4) and high acute illness scores (median national early warning score (NEWS) =7; IQR 5–10) on admission. They had high levels of inflammation mean C-reactive protein 180 mg/L (SD 134.8) and a mean peripheral neutrophil count of 13.1 (SD 5.5) ×10^9^ at admission. 30-day mortality was 36% in participants with CAP and 42% of survivors were readmitted within 90 days (online supplemental table 2).

### Neutrophils from donors with CAP-associated sepsis migrate less accurately to interleukin 8

**Figure 1:**
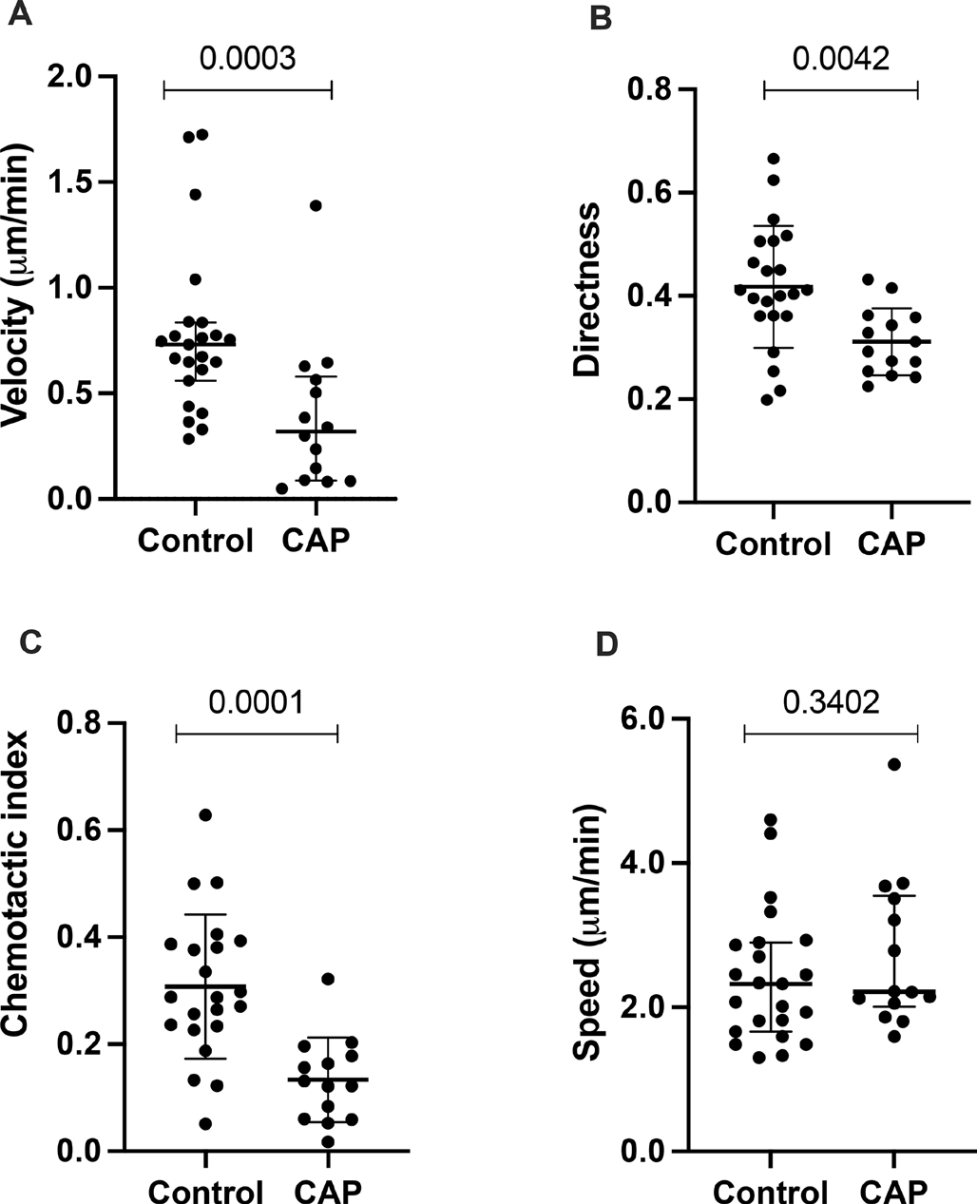
Neutrophils from participants with CAP-associated sepsis have impaired accuracy towards CXCL8. (**A**) Neutrophil velocity is measured in µM/min towards CXCL8. Data shown are median and IQR (p=0.0003). (**B**) Neutrophil directness is a measure of the straightness of the path taken where 1 is a direct straight line over the entire time-lapse. Data presented are mean and SD (p=0.0042). (**C**) Neutrophil Chemotactic Index is a measure of directional accuracy where 1 is direct migration to the chemoattractant and −1 is directly away, data shown are mean and SD, p=0.0001 (**D**) Neutrophil speed in any direction was maintained between groups, data shown are median and IQR (p=0.3402). Data are dot per participant (CAP=14, control=23). CAP, community-acquired pneumonia.

Neutrophil migratory paths showed that cells from CAP donors migrated less accurately towards interleukin 8 (CXCL8) than controls (figure 1). Chemotactic index was reduced in CAP (mean (SD), 0.14 (0.03) in CAP vs 0.31 (0.13) in controls; p<0.0001). Velocity was reduced in CAP donors (median (IQR), 0.32 (0.01–0.58) µm/min in CAP vs 0.73 (0.56–0.83) µm/min; p=0.003). Neutrophil directness was reduced in CAP donors (mean (SD), 0.31 (0.06) in CAP vs 0.41 (0.12) in controls; p=0.0042). Neutrophil speed was maintained in CAP (median (IQR) 2.2 (IQR 2.0–3.6) µm/min compared with 2.3 (1.7–2.9) µm/min for controls; p=0.3402) as summarised in figure 1.

### Viability of isolated neutrophils is reduced in CAP-associated sepsis

**Figure 2:**
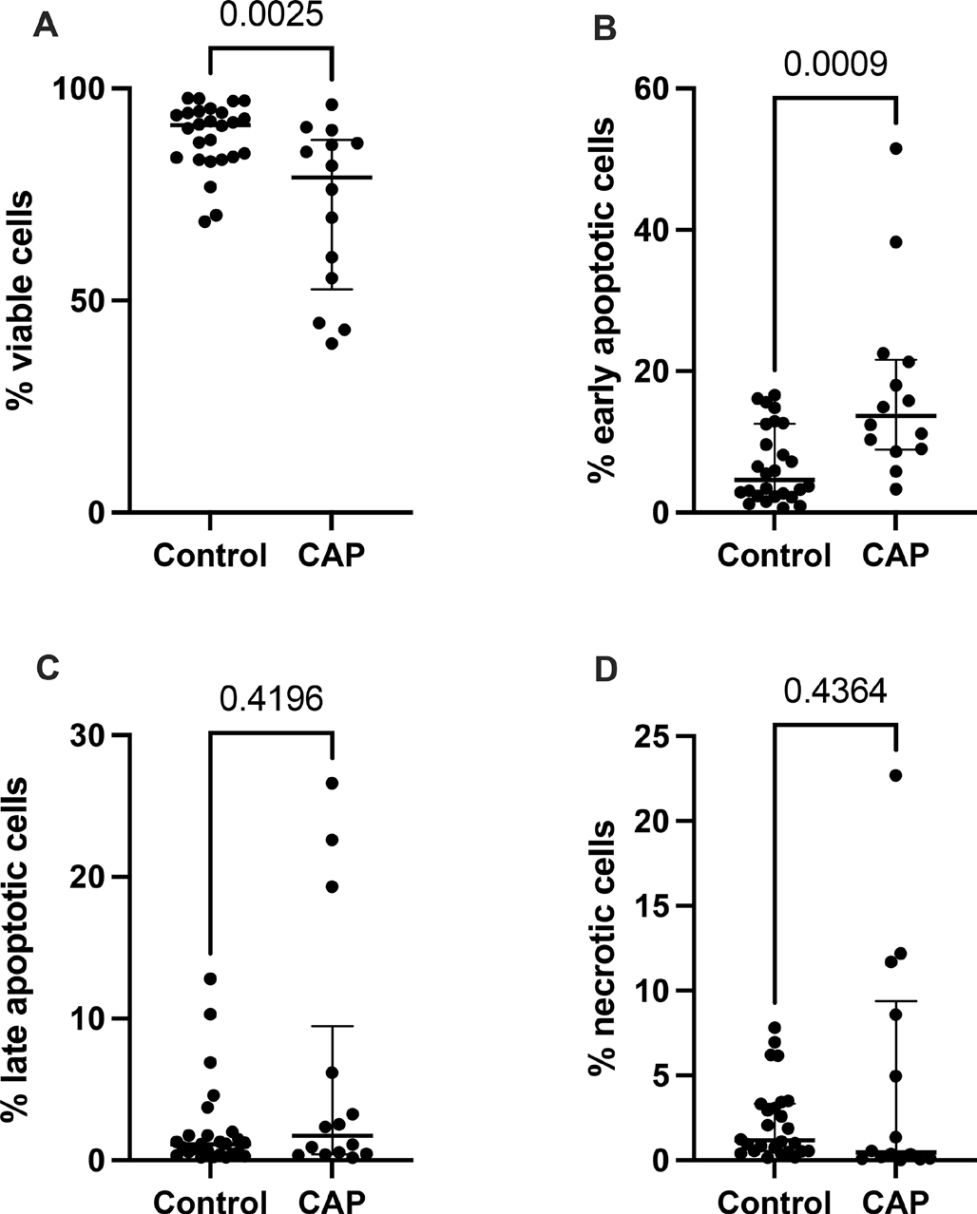
Neutrophil viability in CAP-associated sepsis. (**A**) Viable neutrophils were deemed to be AnV−/PI−. (**B**) Early apoptotic neutrophils were AnV+/PI. (**C**) Late apoptotic cells were AnV+/PI+. (**D**) Necrotic neutrophils were AnV−/PI+. Each point represents data from a single participant (CAP=15, controls=26). Data were not normally distributed (Shapiro-Wilk), and Mann-Whitney tests were performed to determine significance. Horizontal data bars are median with vertical IQR. AnV, Annexin V; CAP, community-acquired pneumonia; PI, propidium iodide.

Neutrophil viability was assessed by flow cytometry using Annexin V and propidium iodide staining (figure 2). Systemic neutrophils from CAP donors exhibited reduced viability and increased early apoptosis (13.7% (IQR 12.7) vs 4.6% (IQR 10.2), p=0.0009). There were no significant differences in the proportion of cells in late apoptosis or necrotic cells between CAP and control donors.

### Neutrophil degranulation and proteinase activity are increased in people with CAP-associated sepsis

**Figure 3:**
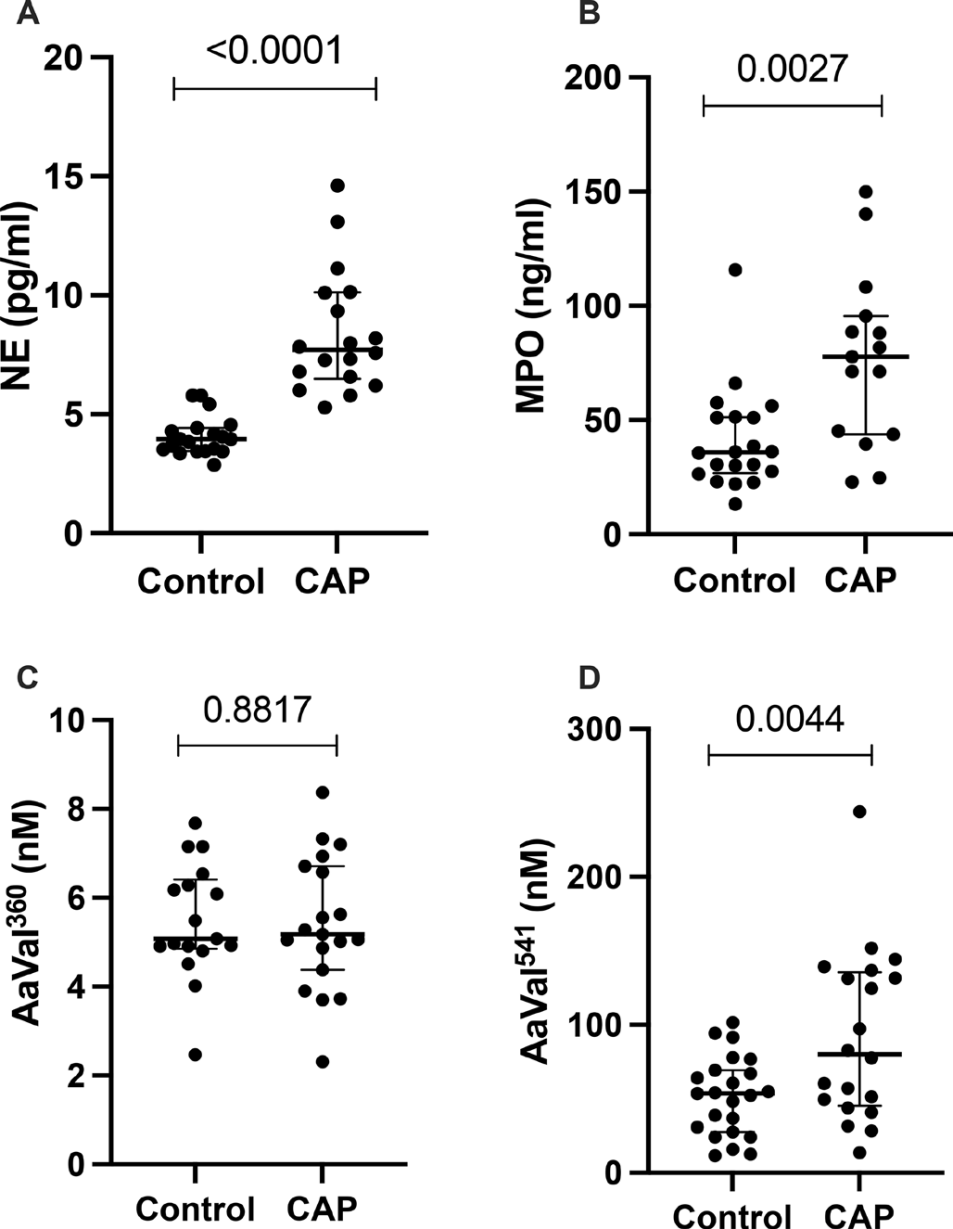
Neutrophil degranulation is increased in CAP-associated sepsis. Plasma neutrophil elastase (NE), Myeloperoxidase (MPO). (**A**) NE was significantly increased in CAP donors (median (IQR) 7.71 (6.4–10.1) ng/mL vs 3.96 (3.46–4.42) ng/mL; p<0.0001) by Mann-Whitney. (**B**) MPO was significantly higher in CAP sample (median (IQR) 87.1 (53.5–147.6) ng/mL vs 37.5 (27.5–53.5) ng/mL; p= <0.0027) by Mann-Whitney. (**C**) Aα-VAL360 was not significantly different. (**D**) Aα-VAL^541^ was significantly higher in CAP (mean (SD) 91.93 (57.44) nM in CAP vs 51.7 (26.46); p=0.0044 by t-test). Data are dots per participant (CAP=20, controls=23). CAP, community-acquired pneumonia.

Plasma NE was significantly increased in CAP donors (median (IQR) 7.71 (6.4–10.1) ng/mL vs 3.96 (3.46–4.42) ng/mL for controls; p<0.0001) (figure 3). MPO was significantly higher in CAP (median (IQR) 87.1 (53.5–147.6) ng/mL vs 37.5 (27.5–53.5)ng/mL; p=0.0027). Further, systemic neutrophil proteinase footprint activity was assessed by measurement of Aα-VAL^541^ (PR3-specific cleavage product) and Aα-VAL^360^ (NE-specific fibrinogen cleavage product). Aα-VAL^541^ was higher in CAP donors than controls (mean (SD) 91.93 (57.44) nM in CAP vs 51.7 (26.46); p=0.0044). However, Aα-VAL^360^ was not significantly different between groups (mean (SD) 5.41 (1.5) nM in CAP vs 5.48 (1.3) nM; p=0.8817).

### Neutrophil oxidative burst in CAP-associated sepsis is reduced in response to PMA

**Figure 4:**
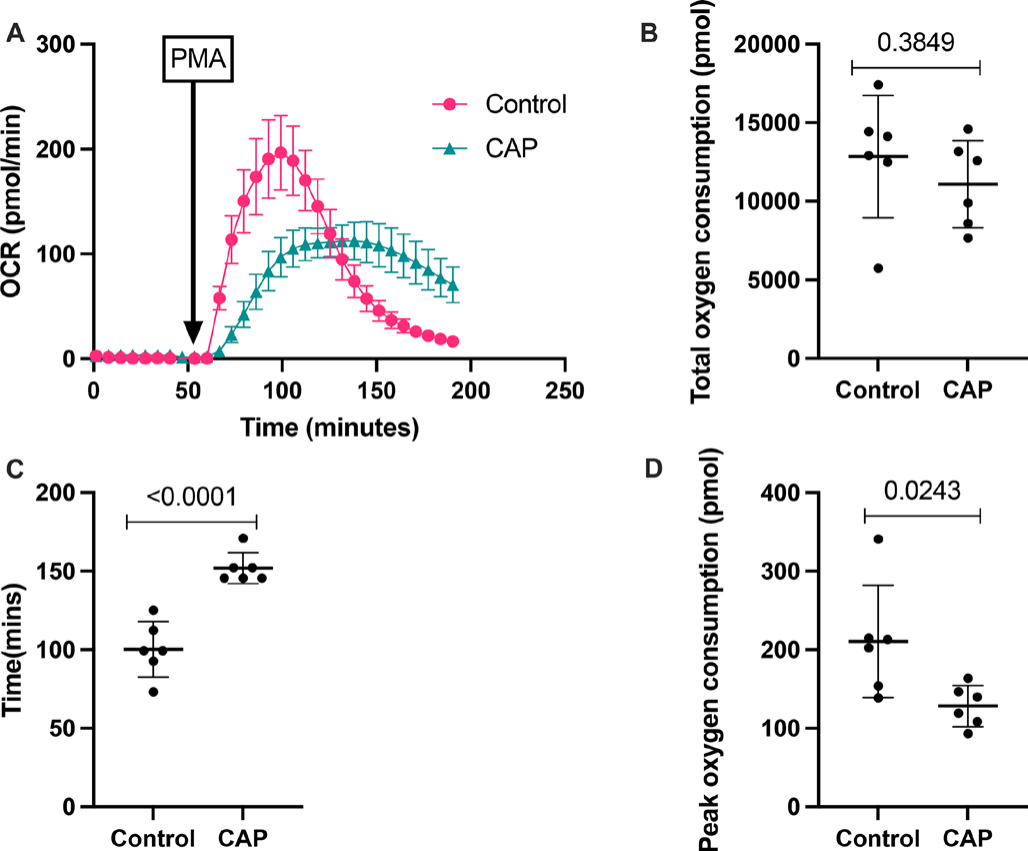
Neutrophil oxygen consumption following PMA stimulation. (**A**) Oxygen consumption over time. (**B**) Total oxygen consumption in response to PMA was unchanged between CAP and controls. (**C**) Time to maximum oxygen consumption was significantly slower in CAP donors (p<0.0001). (**D**) Peak oxygen consumption was significantly lower in CAP (p=0.0243). Time to max OCR, maximum OCR and total oxygen consumption were calculated from the area under the curve analysis. Data represent a point for each participant where this was measured (n=6). Data presented are shown as mean (represented by a horizontal bar) with SD bar lines. T-test used for statistical analysis. CAP, community-acquired pneumonia; OCR, oxygen consumption rate; PMA, phorbol 12-myristate 13-acetate.

Neutrophils from CAP donors consumed similar levels of oxygen as controls (mean (SD), 11 084 (2768) pmol in CAP vs 12 855 (3890) pmol in controls; p=0.3849). However, the kinetics and magnitude of oxygen uptake were significantly different (figure 4). The peak rate of oxygen consumption was higher in the control group (mean (SD) 210.6 (71.33) pmol vs 128.4 (26.15) pmol in CAP; p=0.0243), and time to reach peak oxygen consumption was significantly faster in controls (mean (SD) 100 (17.62) min vs 151 (9.83) min; p<0.0001).

### Neutrophils from CAP-associated sepsis donors are immature with increased expression of activation and adhesion markers

Neutrophils from CAP donors were immature with a lower percentage of CD66b+/CD10+ cells in CAP compared with controls (median (IQR), CAP 68.0 (47.1–83.1)% vs controls 90.9 (81.2–95.1)% p=0.0025).

There were no significant differences in the per cent of cells expressing adhesion markers (CD11b, CD66b and CD11c), activation markers (CD62L) or CXCL8 receptor. However, the surface expression of CD66b and CD11c was significantly higher in CAP (table 2) reflecting increased activation and adhesion.

**Table 2:** Neutrophils cell surface receptor expression

Cellular marker	Control% cells	CAP% cells	P value	Control MFI	CAP MFI	P value
CD66b+/CD10+, median (IQR)	90.9 (81.2–95.1)	68.0 (47.1–83.1)	**0.025**	NA	NA	NA
CD62L, median (IQR)	86.0 (82.1–89.3)	83.5 (66.5–90.6)	0.516	27.0(25.6–34.8)	24.7 (17.4–31.2)	0.223
CD66b, median (IQR)	98.5 (98.5–99.0)	99.0 (98.1–99.6)	0.362	6.5 (5.0–8.6)	11.0 (9.0–17.4)	**0.004**
CD11b, median (IQR)	98.5 (98.1–99.1)	99.0 (98.2–99.3)	0.460	19.6 (11.6–24.3)	23.2 (17.6–30.3)	0.223
CD11c, median (IQR)	90.8 (70.3–95.8)	94.7 (92.2–97.2)	0.117	2.5 (1.6–3.5)	4.0 (2.6–5.6)	**0.019**
CD11b+/CXCR2 bright, median (IQR)	71.5 (12.9–79.5)	75.4 (39.4–85.4)	0.505	NA	NA	NA
CXCR2, median (IQR)	95.2 (86.3–97.3)	97.3 (93.2–99.7)	0.097	8.0 (3.6–16.8)	10.5 (5.6–15.8)	0.562
CD54, median (IQR)	33.9 (20.6–42.4)	37.0 (26.0–55.9)	0.290	1.9 (0.5–2.2)	2.1 (1.3–3.1)	0.313
CXCR4, median (IQR)	17.4 (8.8–29.2)	10.7 (7.2–17.0)	0.105	0.6 (0.5–0.8)	0.7 (0.4–0.8)	0.390
PD-L1, median (IQR)	15.6 (13.6–20.5)	14.8 (9.3–27.5)	0.910	161.0 (106.0–374.0)	249.0 (193.5–670.8)	0.252

Antibodies to detect cluster of differentiation (CD) 11b, CD66b, CD10, CXC chemokine receptor (CXCR) 2, CD11c, CD62L, PD-L1 and CD54 with isotype controls were incubated with isolated neutrophils. Data were acquired using a MACSQuant 10 flow cytometer (Miltenyli Biotec) and analysed using FlowJo software. Mann-Whitney test was used for statistical comparisons. CAP n=19, controls n=9. P-values <0.05 are shown in bold.

CAPcommunity-acquired pneumoniaMFI, median fluorescence intensity; NA, MFI of a single marker not applicable as they are dual marker groups

### Basal and stimulated rates of glycolysis are unchanged in CAP-associated sepsis from age-matched donors but are increased compared with healthy young donors

**Figure 5:**
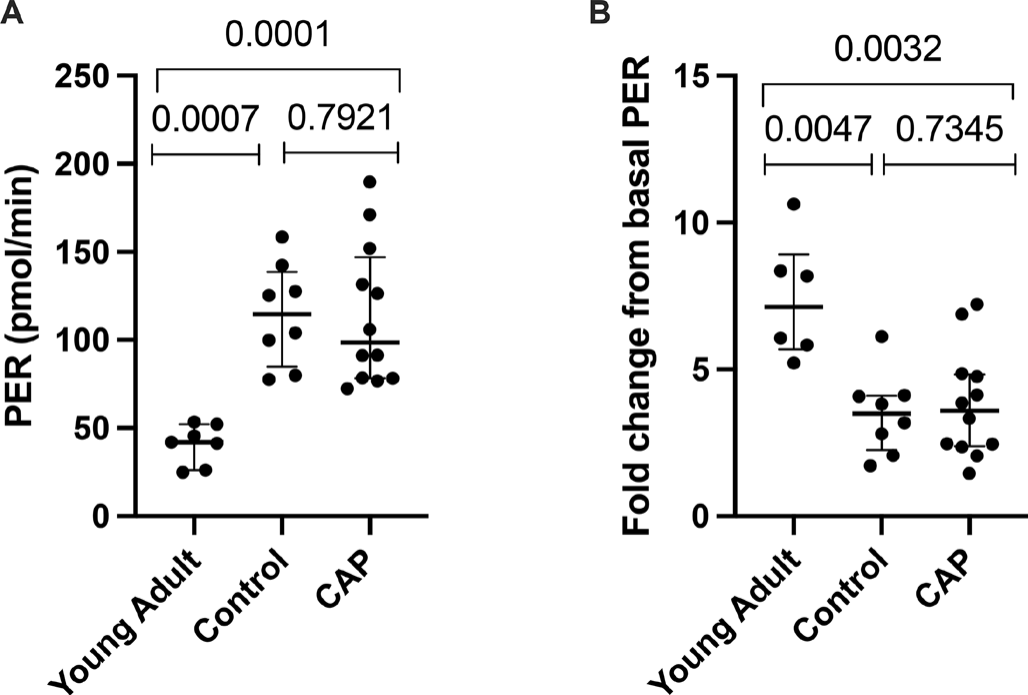
Neutrophil PER is significantly different in CAP-associated sepsis and controls compared with younger adults. (**A**) Basal PER is unchanged in CAP compared with controls (p=0.7921); however, basal PER is significantly lower in young adults compared with controls (p=0.0007) and CAP donors (p=0.0001) (**B**) Fold change in PER after PMA injection is unchanged between CAP and controls (p=0.7345), fold change in PER after stimulation is significantly higher in young adults (p=0.0047) compared with controls and CAP donors (p=0.0032). CAP n=12, control n=8 and healthy young adults n=6. Each point represents a single subject with median and IQR, Mann-Whitney test was used for analysis. Healthy young adults had a median age of 24 years and were healthy with no comorbid condition, and 4/6 were female. CAP, community-acquired pneumonia; PMR, phorbol 12-myristate 13-acetate; PER, proton efflux rate.

Given the altered effector function demonstrated and previous evidence suggesting inhibition of glycolysis abrogates function, we sought to establish whether neutrophil glycolysis was altered in CAP using extracellular flux. There was no significant difference in basal PER (mean (SD) 113.8 (40.05) pmol/min in CAP vs 114.4 (28.96) pmol/min in age-matched controls; p=0.7921). There was also no significant difference in fold change in PER following stimulation with 160 nM PMA (mean (SD) 3.8 (1.8) in CAP vs 3.4 (1.4); p=0.7345) (figure 5).

Glycolysis was also assessed in healthy young donors. Basal PER was significantly lower in healthy young adults compared with CAP and older adult controls (mean (SD)) 40.82 (11.45) pmol/min in young adults vs 113.8 (40.05) pmol/min in CAP vs 114.4 (28.96) pmol/min in controls; p=0.0007). Glycolytic rate was significantly increased following PMA stimulation in healthy young adults (mean (SD)) 7.4 (2.1 vs 3.8 (1.9) in CAP vs 3.5 (1.4) in controls; p=0.0047).

### RNA expression of glycolytic and pentose phosphate pathway enzymes is unchanged

To further investigate neutrophil metabolism during CAP, we performed RNA-sequencing in a subset of participants (n=7 CAP and seven non-CAP controls). Sequencing returned 76M reads per sample, and the raw data met quality control standards, the full dataset is available.^33^ There were 760 differentially expressed genes (absolute fold change ≥0.5 and false discovery rate <0.05). Differential expression is shown in online supplemental figure S4.

There were no significant differences in RNA expression of glycolytic or pentose phosphate pathway enzymes in CAP or control donors (table 3). Pathway analysis performed using Gene Set Enrichment Analysis did not demonstrate any significantly differentially expressed pathways.

**Table 3:** RNA expression of key metabolic enzymes assessed by RNA sequencing

Gene	Gene ID	log2 (FC)	P-adj	P value
Glycolytic enzymes				
Hexokinase	HK2	−0.273	0.939	0.699
Glucose transporter 1	SLC2A1	1.153	0.795	0.398
Pyruvate kinase	PKM	1.353	0.395	0.084
Phosphofructokinase	PFKM	2.562	NA	0.148
Lactate dehydrogenase	LDHA	1.083	0.544	0.162
Monocarboxylate transporter 4	SLC16A3	0.277	0.902	0.584
Glucose-6-phosphate isomerase	GPI	0.427	0.953	0.753
Fructose bisphosphate aldolase	ALDOAP1	0.522	NA	0.815
Phosphoglycerate kinase	PGK1	−0.286	0.812	0.419
Phosphoglycerate mutase	PGAM1	−0.187	0.949	0.739
Enolase	ENO2	−0.300	NA	0.897
Triosephosphate isomerase 1	TPI1	−0.429	0.602	0.910
Bisphosphoglycerate mutase	BPGM	0.394	0.797	0.962
Oxidative pentose phosphate pathway enzymes				
Glucose-6-phosphate dehydrogenase	G6PD	0.99	0.365	0.842
6-Phosphogluconolactonase	PGLS	−0.319	0.859	0.991
Phosphogluconate dehydrogenase	PDG	1.159	0.236	0.028

Differential expression data was acquired using DESeq2Data from 7 CAP and 7seven non-CAP controls. , log2 change, P-adj=p value adjusted for multiple comparisons using Benjamini and Hochberg procedure as described in Love *et al*.^32^ NA=genes with low normalised counts are filtered from Pp-adjusted calculations by DESeq2.^32^

IDidentitylog2 (FC)log 2-fold change

## Discussion

This study provides the first report of neutrophil metabolism in older adults with CAP, demonstrating impaired migratory accuracy and respiratory burst but maintained glycolysis assessed by extracellular flux. Further, there were no significant changes in RNA expression of key glycolytic enzymes individually or at pathway level supporting the extracellular flux analysis. We hypothesised that as neutrophil effector functions are energy intensive and inhibition of glycolysis abolishes neutrophil effector functions,^34^ impaired function in CAP was related to the failure of energy generation by glycolysis.

Overall, these data suggests impaired glycolysis is not linked to the neutrophil dysfunction observed in older adults with CAP.

These data in frail older adults with CAP-associated sepsis contrast to other studies in younger cohorts with milder infections.^19 20 35^ Schuurman *et al* and Pan *et al* identified increased glycolysis in neutrophil transcriptome and metabolome without directly assessing cellular energetics.^19 20^ Borella *et al* used extracellular flux analysis of neutrophils during COVID-19 infection to demonstrate increased glycolysis which was also confirmed using RNAseq.^35^ Important considerations for comparing these studies are differing age, comorbidity, the severity of illness and type of acute illness all of which are known to influence neutrophil function and metabolism, making direct comparisons challenging. For example, Schuurman *et al* studied neutrophil glycolysis using RNA-seq in younger adults with mild CAP and demonstrated increased expression of glycolytic enzymes, but they also enrolled participants with CAP with significantly higher rates of COPD than in the control group,^20^ COPD influences both neutrophil function and metabolic status.^18 36^

We demonstrated that neutrophil metabolism is significantly different in healthy younger adults, with lower basal glycolysis but increased glycolysis in response to PMA stimulation. These data suggest that although neutrophil metabolism changes with age, older adults with acute respiratory infection do not have altered neutrophil glycolysis compared with age-matched controls. This demonstrates the importance of defining the cohort at risk and investigating immune cell function compared with closely matched controls. Despite efforts to match participants for frailty, the increased rates of domiciliary care and reduced mobility in the CAP cohort suggest they are a more vulnerable population than the control group. Frail older adults are at the greatest risk of developing CAP and experience the poorest outcomes in CAP^4^ and act as the ideal control for those with CAP, but this is challenging in practice.

We show increased degranulation evidenced by measurement of total NE and MPO in plasma. We show that PR3 activity is increased in CAP, but despite increased NE in plasma, NE activity is not increased. This is an important observation in neutrophil biology where typically plasma NE levels are reported and assumed to be associated with increased tissue damage. This is a flawed assumption. Here, we report a proteinase footprint assay as a marker of proteinase activity within the lung interstitium,^37^ a direct measure of neutrophil proteinase-derived damage. In alpha-1 antitrypsin (AAT) sufficient individuals, systemic levels of AAT will result in rapid inhibition of serine proteinases preventing fibrinogen cleavage in the blood compartment; however, in the interstitium, AAT levels are much lower therefore unregulated proteinase activity results in fibrinogen cleavage. These cleavage products can then be detected in plasma.

These data suggest that despite the significant increase in degranulation in patients with CAP, NE released within the interstitium is well controlled by AAT (an acute phase diffusible protein, especially in lung inflammation), yet PR3 activity within the interstitium is significantly increased. Potential explanations, not tested here, for the differential footprint activity levels of PR3 compared with NE is attributed to a combination of; (a) differential inhibition of NE by AAT in CAP as NE has a much higher affinity for AAT than PR3,^38^ (b) NE can be inhibited by organisms which commonly cause pneumonia^39^ and finally PR3 is more abundant and shed more rapidly from the cell surface so may overwhelm the local concentration of serine proteinase inhibitors, thus allowing unrestricted proteinase activity.^40^

This study uses a total oxygen consumption assay to determine neutrophil oxidative burst following stimulation with PMA. This provides advantages over the typical chemiluminescent assays which are highly selective for individual species of reactive oxygen and suffer from artefacts related to redox recycling.^41^ Our study shows similar levels of overall oxygen consumption but significant differences in time taken for oxygen consumption and peak of oxygen consumption. This demonstrates the importance of considering the kinetics of neutrophil responses. Due to their potentially toxic capacity, the ideal neutrophil oxidative burst would reach a rapid peak and quickly wane, in contrast we demonstrate a slow burn of oxidative burst.

There are limitations to our study. We only assessed glycolysis; in previous work, we and others have been unable to assess rates of mitochondrial respiration in neutrophils accurately using extracellular flux.^30 42^ This is in keeping with the low density of mitochondria in neutrophils and evidence demonstrating that mitochondrial function does not contribute to overall ATP balance.^18^ In addition, we did not directly examine shifts towards the pentose phosphate pathway which has been shown to be a key regulator of ROS and therefore NETosis^43^; however, enzymes measured by RNAseq involved in the pentose phosphate pathway were not altered at individual or pathway level.

We assessed the metabolism and RNA expression of glycolytic enzymes but did not measure cellular ATP or glycogen stores. This is therefore only a snapshot of neutrophil function and metabolism. An optimal response would be a rapid and robust response early in the disease course, with rapid waning to allow for resolution of inflammation, and should be a feature of future studies. The inability to detect a significant difference in glycolysis by direct measurement, or in the transcriptome could be due to low sample size. However, based on the data presented here, 48 000 participants would be needed in each arm to detect a significant difference in glycolysis in CAP versus age-matched controls.

Participants with CAP were recruited 3–4 days after symptom onset meaning the initial changes in glycolysis may have occurred early and resolved to prevent overactivation and tissue damage by the time of study recruitment. Understanding this would require large numbers of community-dwelling participants with longitudinal sampling prior to the onset of respiratory infection.

The relationship of neutrophil maturity and metabolism is unknown in humans; however, in murine studies, mitochondrial respiration appears to play a role in bone marrow differentiation,^44^ suggesting that the immature neutrophils identified in this study in participants with CAP may use metabolic pathways other than glycolysis in preference.

We only recruited outside of the critical care setting, and we used a pragmatic tool to screen for sepsis (qSOFA) providing a surrogate definition. qSOFA was the recommended tool for sepsis screening at the inception of this study^25^ but is no longer recommended.^45^ Participants were predominantly of white British ethnicity which may limit generalisability to other populations. The chemotaxis, degranulation, oxidative burst, cell surface marker and glycolysis data are not corrected for multiple comparisons given the low number of planned comparisons.^22^ Finally, we were unable to perform all assays in all donors due to limited cell numbers, and time and staff restrictions in performing all assays on the same day.

Despite these limitations, there are important findings that warrant further investigation, particularly the difference seen in glycolytic metabolism between young and aged donors. There is increasing literature investigating immunosenescence, and the data presented here suggest that alterations in metabolism may play a role in altered function with age. Our study recruited a pragmatic population of participants with CAP and well-matched controls. This is a disease-prone population but poorly studied. We confirm that glycolytic enzymes are not significantly different at the transcript level, supporting the extracellular flux data demonstrating glycolysis is unlikely to contribute to abnormal effector function in CAP. There has been increasing interest in targeting immunometabolism in sepsis, our data provide caution that each patient population should be carefully phenotyped with consideration for known factors which influence immune cell function.

## supplementary material

10.1136/thorax-2024-222215 online supplemental file 1

## Data Availability

Data are available in a public, open access repository. Data are available upon reasonable request.
