# Supplementary material for: Hospitalised older adults with community-acquired pneumonia and sepsis have dysregulated neutrophil function but preserved glycolysis
Source: Thorax. 2024 Dec 16;80(2):e222215. doi: 10.1136/thorax-2024-222215 (PMC11877105; doi:10.1136/thorax-2024-222215)
Supplement: online supplemental file 1 [file thorax-80-2-s001.pdf]

Hospitalised older adults with community acquired pneumonia and sepsis have dysregulated neutrophil function but preserved glycolysis

Supplementary Data

Methods

Neutrophil Viability

Isolated neutrophils were resuspended in Annexin V (AnV) binding buffer (diluted 10X buffer solution 0.2M HEPES, 1.4M NaCl and 25mM $CaCl_2$ 1:10 (v/v) in dH_2O) at $1x10^6$/ml and kept on ice for assay. AnV FITC stain (Miltenyl Biotec, 130-093-060) was added at 1:500 dilution in AnV binding buffer and incubated on ice in the dark for twenty minutes. Neutrophils were washed twice (250x *g,* 10 minutes, 4°C) in AnV binding buffer and then suspended in 200µl AnV binding buffer. Immediately prior to data acquisition propidium iodide (PI) (Sigma-Aldrich, Poole UK, product no. P4864) was added 1:100 (v/v). Samples were analysed on MACSquant Analyzer 10. Fluidics set to medium and 10,000 neutrophil events gated on forward scatter (FSC) and side scatter (SSC). Gates for positive events were plotted compared to unstained control neutrophils. Proportions of live (viable), early apoptotic, late apoptotic, and necrotic cells were determined based on gating. Viable were determined as AnV-/PI-, early apoptotic were AnV+/PI-, late apoptotic were AnV+/PI+ and necrotic were AnV-/PI+.

Participant recruitment

Supplementary Table 1: Study Inclusion and exclusion criteria

CAP Cohort	
Inclusion Criteria	**Exclusion criteria**
Community acquired pneumonia and sepsis admitted to hospital<br>Age ≥65 years	Asthma<br>Chronic obstructive pulmonary disease<br>Metastatic malignancy<br>Haematological malignancy<br>Participant already enrolled in study of novel/unlicensed treatment<br>Immunosuppressive treatment in preceding 12 weeks<br>Known immunodeficiency syndrome

	Treatment withdrawal imminent<br>Current infection with COVID-19
Control Cohort	
Inclusion Criteria	**Exclusion criteria**
Age ≥65 years or ≤35 years	As above plus:<br>Acute illness in preceding 12 weeks<br>Unable to give informed consent to enrolment

Eligible patients were recruited within 36 hours of hospitalisation following written informed consent, or consultee agreement from personal or professional legal representatives. Retrospective consent was obtained when possible.

CAP participant recruitment is shown in Figure S1

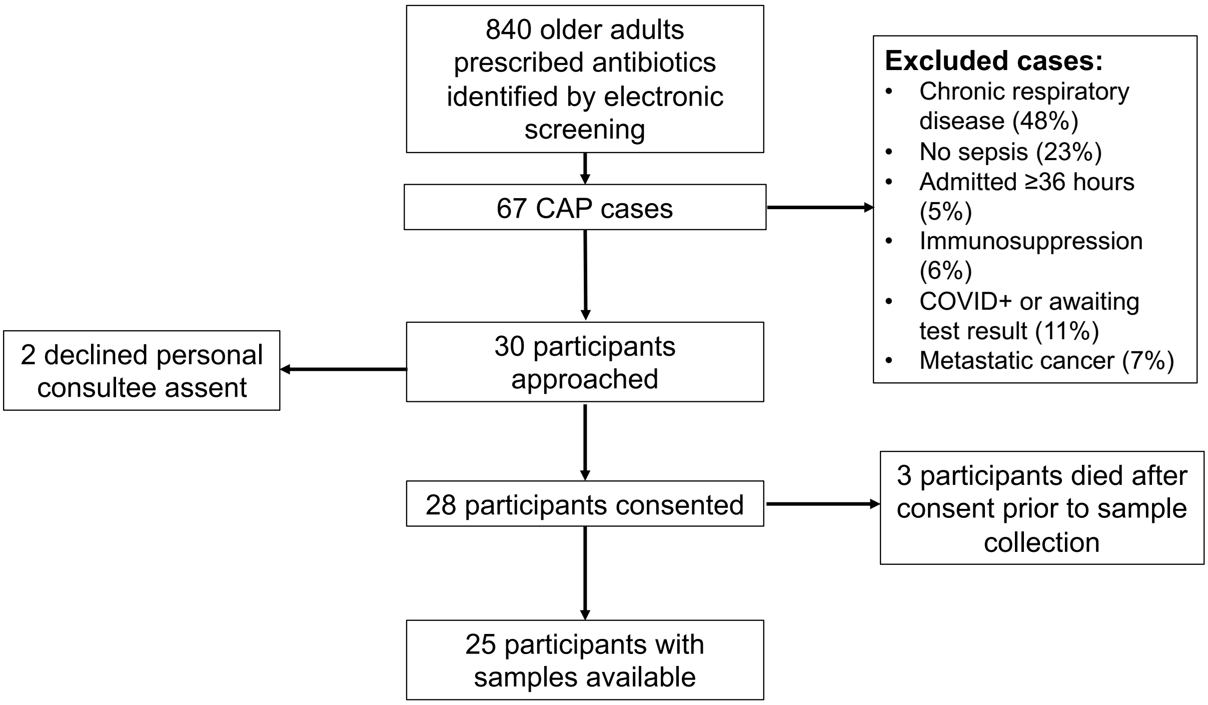

Figure S1: Consort Diagram describing CAP participant recruitment

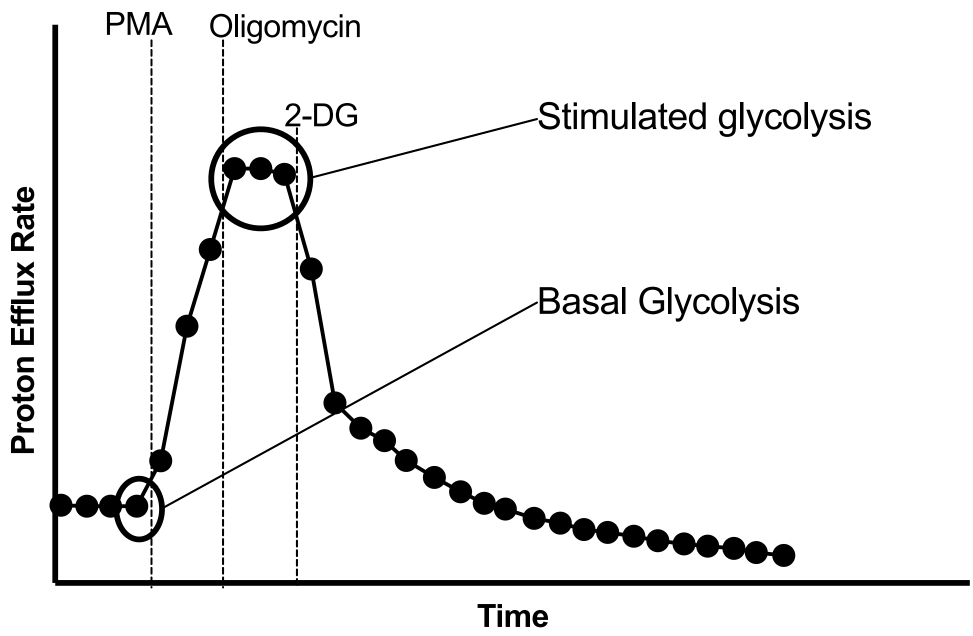

Figure S2: Injection strategy and parameters used in assessment of neutrophil glycolysis.
Basal glycolysis was the PER reading taken immediately prior to PMA injection, stimulated glycolysis was the mean PER reading of three measurements taken between oligomycin and 2-deoxyglucose measurements.

RNA Data Processing

Data processing utilized the Galaxy platform (1) with FastQC and Cutadapt for adapter trimming (2) and RNA STAR for alignment to the human genome GRCh38/hg38 (3). Gene counts were derived via FeatureCounts (4) with DESeq2 assessing differential expression (5). Pathway analysis employed Gene Set Enrichment Analysis (GSEA) against KEGG and GO databases (6–8) applying Benjamini-Hochberg correction for multiple comparisons. Visualization was conducted in R (v4.3.1, R Foundation for Statistical Computing, 2021). Analysis pipeline is shown in Figure S3.

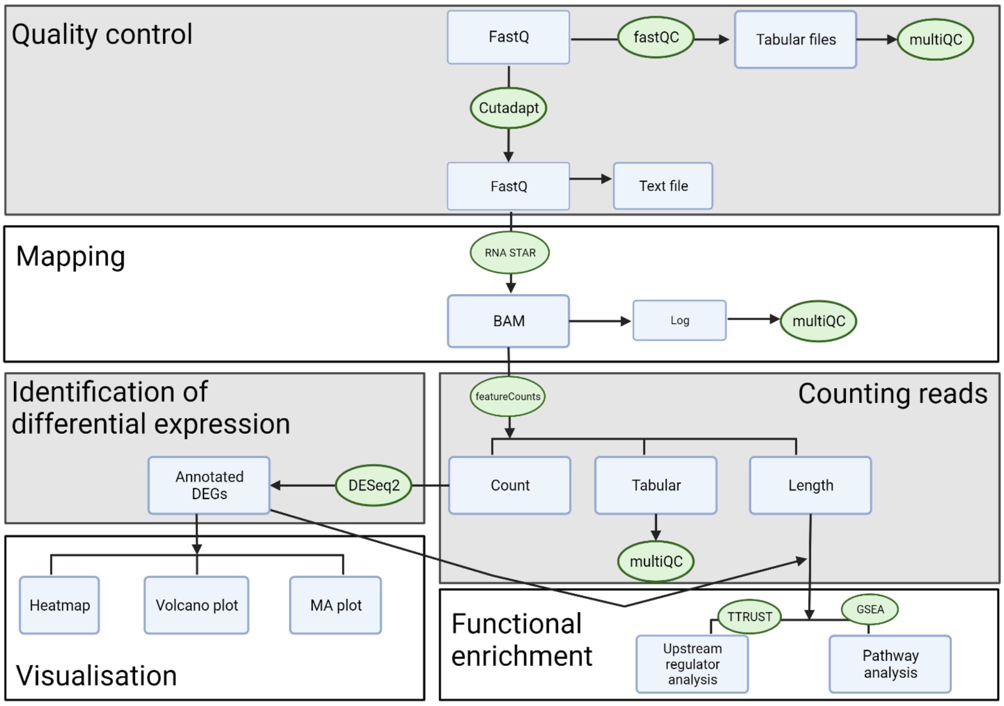

Figure S3: RNA Analysis Pipeline
Raw sequencing files in .fastQ form were uploaded to galaxy web server, where fastQC and cutadapt were used to trim sequences of adapter reads and ensure quality control (QC). Each read was then mapped using RNA STAR, further QC was performed using multiQC. After mapping gene expression was counted using feature counts. This expression data was then used in differential expression analysis using DESeq2 and to normalise differentially expressed genes (DEGs) for functional enrichment. Outputs of DESeq2 were used for data visualisation and functional enrichment. Blue boxes are file types or outputs, green ellipses are tools used to analyse data. Quality control, mapping, read counts and identification of DEGs was completed using galaxy server. Visualisation was completed using R. Upstream regulator analysis was completed using searchlight 2 and pathway analysis was completed using GSEA. Created using biorender.com.

Results

Supplementary Table 2: Pneumonia severity indices and outcomes

Pneumonia severity factors	
Median CURB65 score (IQR)	3 (3-4)
Median NEWS2 score (IQR)	7 (5-9.5)
Median qSOFA score (IQR)	2 (2-2)
Median SOFA score (IQR)	2.5 (2-4)
Mean white cell count (x10^9) (S.D)	15.1 (6.1)
Mean neutrophil count (x10^9) (S.D)	13.1 (5.5)
Mean CRP (mg/L) (S.D)	180 (134.8)
Mean Creatinine (mmol/L) (S.D)	88.4 (37.5)
Mean Urea (mmol/L) (S.D)	9.54 (4.8)
Mean ALT (units/L) (S.D)	31.4 (26.2)
Mean Lactate (mmol/L) (S.D)	2.7 (1.2)
Mean Respiratory rate/min (S.D)	25 (6)

Mean systolic blood pressure (mmHg) (S.D)	117 (26)
Mean diastolic blood pressure (mmHg) (S.D)	66 (17)
Mean heart rate/min (S.D)	102 (21)
Viral throat swab performed (%)	25 (100)
Sputum sample performed (%)	2 (8)
Blood culture performed (%)	14 (56)
Outcomes	
Inpatient mortality; number (%)	6 (24)
30d mortality; number (%)	9 (36)
90d mortality; number (%)	10 (40)
ICU admission; number (%)	2 (8)
90d readmission if survived index admission; number (%)	8 (42)
Increased care needs at discharge; number (%)	9 (47)
Median LOS for survivors (IQR)	10 (5-17)

Blood results and observations are those from admission. IQR=interquartile range, NEWS2= national early warning score 2, qSOFA= quick sequential organ failure assessment score, SOFA= sequential organ failure assessment score, S.D=standard deviation, CRP= c reactive protein, ALT= alanine transaminase, LoS= length of stay, mmHg=millimetres of mercury. N=25 CAP participants.

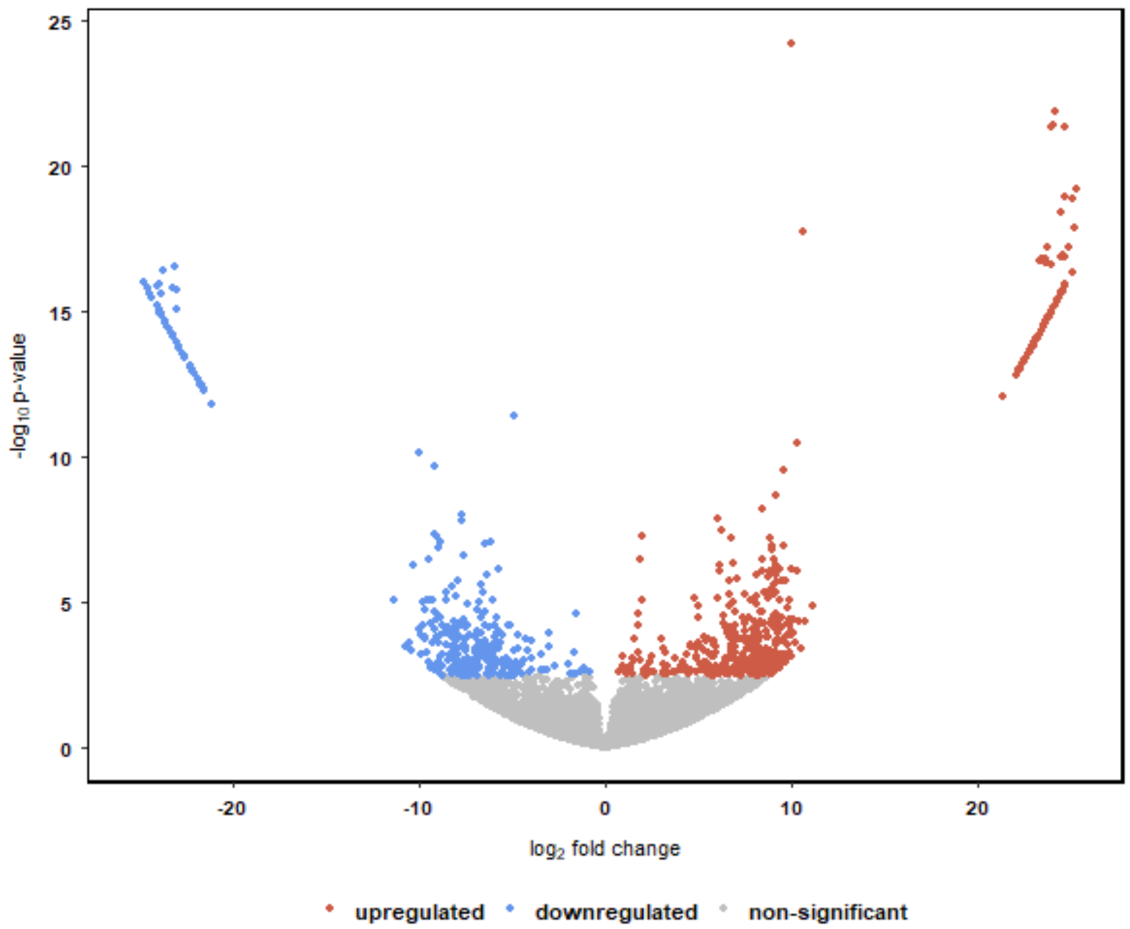

Figure S4: Volcano plot demonstrating differential expression of genes.

Volcano plot for the comparison between CAP and control. Significantly differential genes (p.adj < 0.05, absolute log2 fold > 0.5) are shown in red (upregulated) or blue (downregulated) and non−significant genes in grey. A positive fold change indicates higher expression in CAP than in control. N= 7 CAP and 7 control.

References

1. Afgan E, Baker D, van den Beek M, Blankenberg D, Bouvier D, Čech M, et al. The Galaxy platform for accessible, reproducible and collaborative biomedical analyses: 2016 update. Nucleic Acids Res [Internet]. 2016 Feb;44(W1):W3–10. Available from: https://pubmed.ncbi.nlm.nih.gov/27137889/
2. Martin M. Cutadapt removes adapter sequences from high-throughput sequencing reads. EMBnet J [Internet]. 2011 Feb;17(1):10–2. Available from: https://journal.embnet.org/index.php/embnetjournal/article/view/200/479
3. Dobin A, Davis CA, Schlesinger F, Drenkow J, Zaleski C, Jha S, et al. STAR: ultrafast universal RNA-seq aligner. Bioinformatics [Internet]. 2013

Feb;29(1):15–21. Available from: https://academic.oup.com/bioinformatics/article/29/1/15/272537
4. Liao Y, Smyth GK, Shi W. featureCounts: an efficient general purpose program for assigning sequence reads to genomic features. Bioinformatics [Internet]. 2014 Feb;30(7):923–30. Available from: https://academic.oup.com/bioinformatics/article/30/7/923/232889
5. Love MI, Huber W, Anders S. Moderated estimation of fold change and dispersion for RNA-seq data with DESeq2. Genome Biol [Internet]. 2014 Feb;15(12):1–21. Available from: https://genomebiology.biomedcentral.com/articles/10.1186/s13059-014-0550-8
6. Subramanian A, Tamayo P, Mootha VK, Mukherjee S, Ebert BL, Gillette MA, et al. Gene set enrichment analysis: A knowledge-based approach for interpreting genome-wide expression profiles. Proceedings of the National Academy of Sciences. 2005 Feb;102(43):15545–50.
7. Consortium GO. Gene Ontology Consortium: going forward. Nucleic Acids Res. 2015 Feb;43(D1):D1049–56.
8. Kanehisa M, Sato Y, Kawashima M, Furumichi M, Tanabe M. KEGG as a reference resource for gene and protein annotation. Nucleic Acids Res. 2016 Feb;44(D1):D457–62.
